# Markers of Immune Activation and Inflammation Are Associated with Higher Levels of Genetically-Intact HIV in HIV-HBV Co-Infected Individuals

**DOI:** 10.1128/jvi.00588-22

**Published:** 2022-08-02

**Authors:** Xiao Qian Wang, Jennifer M. Zerbato, Anchalee Avihingsanon, Katie Fisher, Timothy Schlub, Ajantha Rhodes, Jennifer Audsley, Kasha P. Singh, Wei Zhao, Sharon R. Lewin, Sarah Palmer

**Affiliations:** a Centre for Virus Research, The Westmead Institute of Medical Research, The University of Sydney, Sydney, New South Wales, Australia; b Sydney Medical School, Westmead Clinical School, Faculty of Medicine and Health, The University of Sydney, Sydney, New South Wales, Australia; c Department of Infectious Diseases, University of Melbourne at the Peter Doherty Institute for Infection and Immunity Melbourne, Victoria, Australia; d HIV-NAT, Thai Red Cross AIDS Research Center (TRCARC), Bangkok, Thailand; e Sydney School of Public Health, Faculty of Medicine and Health, University of Sydney, Sydney, New South Wales, Australia; f Victorian Infectious Diseases Service, Royal Melbourne Hospital at the Peter Doherty Institute for Infection and Immunity, Melbourne, Victoria, Australia; g Department of Infectious Diseases, Alfred Hospital and Monash University, Melbourne, Victoria, Australia; Emory University

**Keywords:** full-length sequencing, HIV-HBV co-infection, human immunodeficiency virus, hepatitis B virus, HIV persistence

## Abstract

Co-infection with hepatitis B (HBV) and human immunodeficiency virus (HIV) increases overall and liver-related mortality. In order to identify interactions between these two viruses *in vivo*, full-length HIV proviruses were sequenced from a cohort of HIV-HBV co-infected participants and from a cohort of HIV mono-infected participants recruited from Bangkok, Thailand, both before the initiation of antiretroviral therapy (ART) and after at least 2 years of ART. The co-infected individuals were found to have higher levels of genetically-intact HIV proviruses than did mono-infected individuals pre-therapy. In these co-infected individuals, higher levels of genetically-intact HIV proviruses or proviral genetic-diversity were also associated with higher levels of sCD14 and CXCL10, suggesting that immune activation is linked to more genetically-intact HIV proviruses. Three years of ART decreased the overall level of HIV proviruses, with fewer genetically-intact proviruses being identified in co-infected versus mono-infected individuals. However, ART increased the frequency of certain genetic defects within proviruses and the expansion of identical HIV sequences.

**IMPORTANCE** With the increased availability and efficacy of ART, co-morbidities are now one of the leading causes of death in HIV-positive individuals. One of these co-morbidities is co-infection with HBV. However, co-infections are still relatively understudied, especially in countries where such co-infections are endemic. Furthermore, these countries have different subtypes of HIV circulating than the commonly studied HIV subtype B. We believe that our study serves this understudied niche and provides a novel approach to investigating the impact of HBV co-infection on HIV infection. We examine co-infection at the molecular level in order to investigate indirect associations between the two viruses through their interactions with the immune system. We demonstrate that increased immune inflammation and activation in HBV co-infected individuals is associated with higher HIV viremia and an increased number of genetically-intact HIV proviruses in peripheral blood cells. This leads us to hypothesize that inflammation could be a driver in the increased mortality rate of HIV-HBV co-infected individuals.

## INTRODUCTION

Antiretroviral therapy (ART) has improved the life expectancy of HIV-positive individuals globally, with HIV-positive individuals on suppressive ART being expected to achieve a similar life span to that of the general population (1). Improvements in ART and the availability of HIV and HBV-active drugs, such as tenofovir, have also been integral in achieving good clinical outcomes in HIV-HBV co-infected individuals (2). However, despite this, HIV-HBV co-infected individuals experience increased mortality rates, even when virally suppressed on HBV-active ART, compared to mono-infection (2). HIV-HBV co-infection leading to liver disease and hepatocellular carcinoma (HCC) is one of the major contributors to mortality in HIV-positive individuals (3, 4). It is therefore essential to understand the mechanisms behind these poor outcomes during HIV-HBV co-infection, even when both viruses are suppressed on ART.

Current research efforts have identified that HIV co-infection affects HBV replication. *In vitro* cell culture models of co-infection have demonstrated that HIV co-infection of hepatocytes transfected with HBV express greater levels of HBV surface antigen (HBsAg) but not HBV DNA (5). Co-infected individuals have also been shown to express higher levels of HBsAg than do HBV mono-infected individuals, and they also experience more liver inflammation, even when they are on suppressive HBV-active ART (6). An increase in certain HBV genetic mutations, which is more frequently observed in HIV co-infected individuals, is associated with increased HCC risk (7). When observing the natural courses of infection of these two viruses, there is reasonable evidence to suggest that HIV could impact HBV pathogenesis, as it exacerbates the rate of HBV disease progression. On the other hand, it is unclear whether HBV co-infection affects HIV pathogenesis, as studies show conflicting results as to whether HIV-HBV co-infection exacerbates the rate of progression to AIDS (8–10).

As both HIV and HBV persist despite ART suppression, recent research has focused on whether prolonged immune activation and inflammation may be a contributing factor to the poorer outcomes observed in HIV-HBV co-infected individuals. Recently, it has been found that HIV-HBV co-infected individuals had higher levels of pro-inflammatory cytokines, such as IL-6 and IL-8, compared to HBV mono-infected individuals (11). A study examining the relationship between microbial translocation and liver damage has found elevated levels of the immune activation markers lipopolysaccharide (LPS) and soluble CD14 (sCD14), as well as chemokines, such as CXCL10 and CCL2, in HIV-HBV co-infected individuals compared to HBV mono-infected individuals (12). After the initiation of HBV-active ART, some markers of immune activation were reduced, while others remained elevated (12). This same study also showed an association between increased levels of pro-inflammatory cytokines and increased levels of hepatic injury (12). Two recent studies have drawn comparisons between HIV mono-infected cohorts and HIV-HBV co-infected cohorts, but they present conflicting results as to whether HBV co-infection leads to higher levels of inflammation or immune activation compared to HIV mono-infection (13, 14). These conflicting results are partially due to the different markers measured by these two studies.

Previous studies have shown that most integrated HIV proviruses are genetically defective and are therefore unable to replicate (15–17). This is especially evident in HIV mono-infected individuals on long-term suppressive ART, as these studies have demonstrated, but limited studies have been conducted to genetically characterize the proviruses within cellular samples from participants before ART initiation or participants co-infected with HBV.

It remains uncertain whether HBV affects HIV replication or HIV reservoir formation in CD4 T cells. We focused on bridging this gap in the field, with the hypothesis that HBV co-infection influences HIV replication and reservoir development. To investigate this, we sequenced near full-length HIV proviruses from co-infected individuals to observe associations between HIV proviral characteristics and markers of HBV disease, immune activation, inflammation, and liver damage.

## RESULTS

### ART-naive, HIV-HBV co-infected individuals have high but variable levels of genetically-intact HIV proviruses.

Few studies have conducted full-length HIV sequencing of samples from untreated participants, with none in the context of HBV co-infections (15). We investigated the levels of HIV proviruses and their genetic characteristics in ART-naive, HIV-HBV co-infected individuals. Previous studies have revealed that most of the HIV proviruses that are present during ART are defective. Those that lack these genetic defects are termed “genetically-intact” and are likely to be replication competent. Here, we obtained a total of 1,016 HIV proviruses from the peripheral blood CD4^+^ T cells of 18 ART-naive, HIV-HBV co-infected individuals. In order to determine if these proviruses were intact or defective, they were sequenced using full-length individual proviral sequencing (FLIPS), as described previously (16).

**FIG 1:**
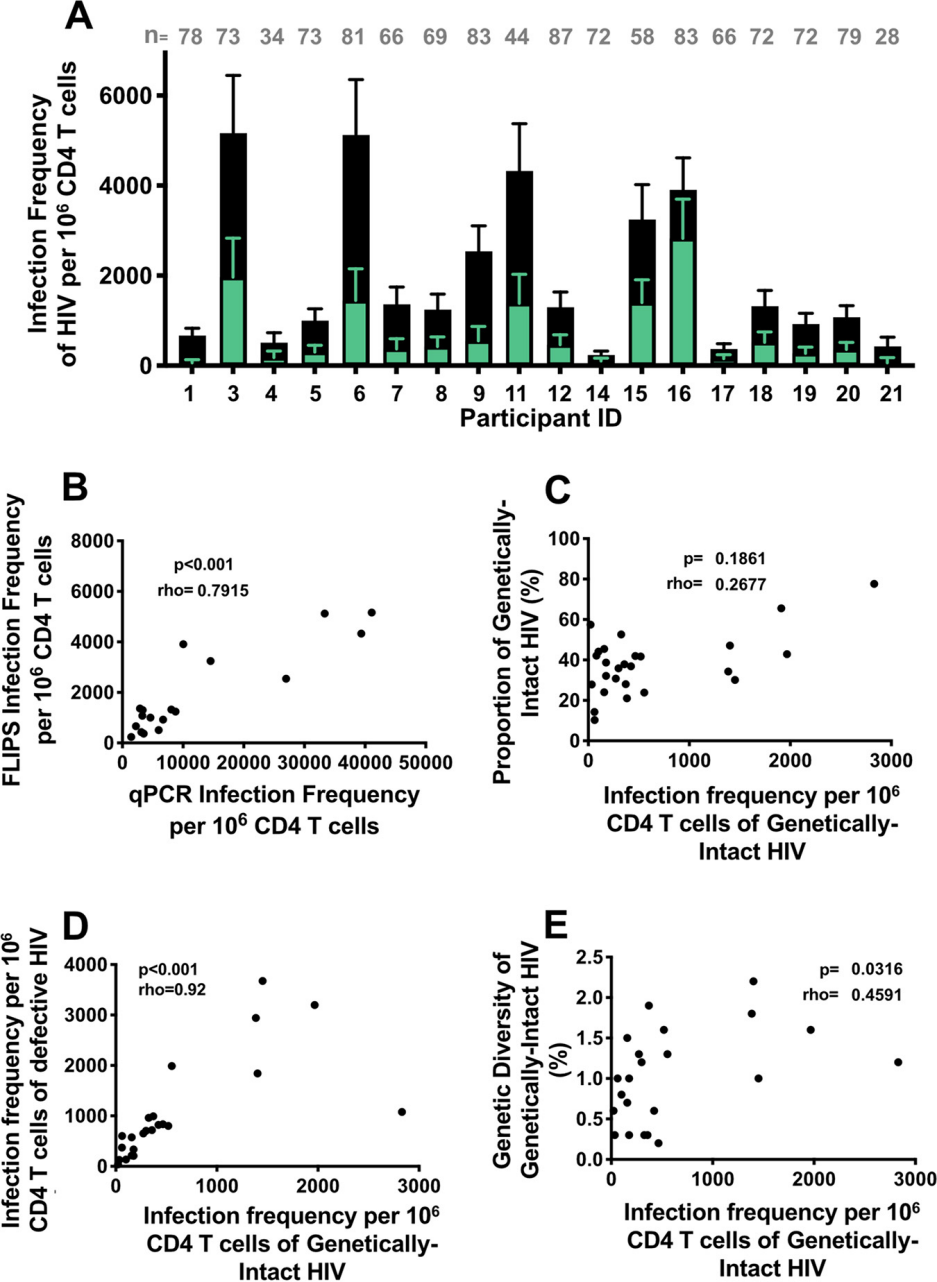
(A) Infection frequency of HIV per 10^6^ CD4^+^ T cells from pre-therapy samples from 18 HIV-HBV co-infected participants, where the frequency of genetically-intact HIV (in green) may also be viewed as a proportion of the total HIV frequency (in black). The number of HIV sequences obtained for each participant is denoted by n. (B) Spearman’s correlation between the infection frequency of the total HIV measured by qPCR and the infection frequency of HIV (genetically-intact and defective) measured by FLIPS. (C) Spearman’s correlation between the infection frequency of HIV proviruses with the proportion of genetically-intact HIV provirus. (D) Spearman’s correlation between the infection frequencies of genetically-intact and defective HIV proviruses. (E) Spearman’s correlation between the infection frequencies of genetically-intact HIV proviruses with the genetic diversity of genetically-intact HIV provirus.

The level of total HIV DNA, used as a surrogate marker for infection frequency, was quantified by two methods. The first method utilized a quantitative polymerase chain reaction (qPCR) assay with primers that target a small region of the HIV LTR. The second method employed FLIPS at the single molecule level, which can establish the infection frequency of HIV proviruses within an individual and can also provide the ability to distinguish between genetically-intact and defective proviruses. Using FLIPS, the infection frequency of the overall HIV proviruses (comprised of both the genetically-intact and the detective) per 10^6^ CD4^+^ T cells and the infection frequency of genetically-intact HIV proviruses per 10^6^ CD4^+^ T cells were calculated for all participants. These co-infected individuals had high and variable levels of both overall and genetically-intact HIV proviruses (range: 200 to 5,000 and 60 to 3,000 copies per 10^6^ CD4^+^ T cells, respectively) (Fig. 1A). The total HIV DNA levels obtained by qPCR (ranging from 260 to 41,000 copies per 10^6^ CD4^+^ T cells) were much higher than the infection frequency of proviruses obtained with the FLIPS assay. However, there was a strong (ρ = 0.79) and significant association between the infection frequency observed using qPCR and that observed using FLIPS (*P* < 0.001) (Fig. 1B).

The proportion of HIV proviruses that were genetically-intact was also variable between participants (Fig. 1A). However, there was no significant association between the infection frequency of genetically-intact HIV proviruses with the proportion of these genetically-intact proviruses (Fig. 1C). Interestingly, there was a strong (ρ = 0.92) and significant correlation between the infection frequency of genetically-intact proviruses and the infection frequency of defective proviruses (*P* < 0.001) (Fig. 1D). This could be due to the high number of genetically-intact proviruses identified during pre-therapy for all participants compared to what is typically observed in participants on long-term ART. Alternatively, the years of untreated infection could also explain the accumulation of defective proviruses over time. We also calculated the genetic diversity (average pairwise distance) of the genetically-intact HIV proviruses using the p-distance model, which determines the genetic differences between each sequence. We found the genetic diversity of the genetically-intact HIV proviruses to be variable between the participants, with an average p-distance of 0.015 (range: 0.002 to 0.022) (Table S1). There was a moderately strong and significant association between the infection frequency of genetically-intact HIV proviruses and the genetic diversity of these genetically-intact proviruses (*P* = 0.03) (Fig. 1E).

### Levels of genetically-intact HIV proviruses are associated with HIV clinical parameters but not HBV clinical parameters in untreated HIV-HBV co-infected participants.

**FIG 2:**
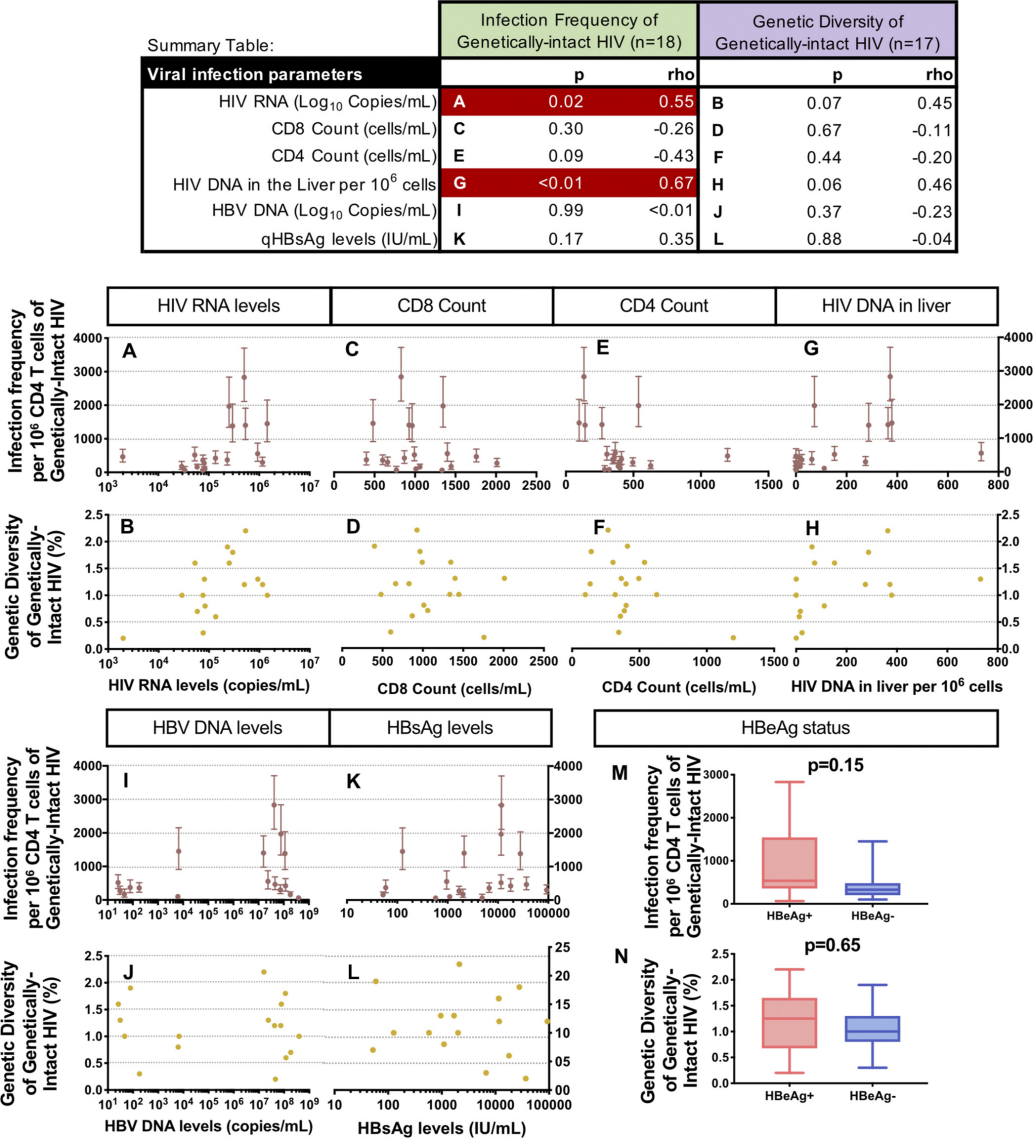
Spearman’s correlation analyses comparing viral parameters with the infection frequency and the genetic diversity of genetically-intact HIV proviruses (Summary Table: significant associations in red). The association between the infection frequency (maroon) and the diversity (yellow) of genetically-intact HIV proviruses and parameters related to HIV infection are shown in Fig. 2A–H. The scale of the *x*-axis changes for each clinical HIV parameter. The association between the infection frequency (maroon) and the diversity (yellow) of genetically-intact HIV proviruses and parameters related to HBV infection are shown in Fig. 2I–L. The *x*-axis changes for each clinical HBV parameter. The effects of positive HBeAg (red; *n* = 10) and negative HBeAg (blue; *n* = 8) status on the infection frequency and the diversity of genetically-intact HIV are shown in Fig. 2M and N.

As the levels of genetically-intact HIV provirus varied across participants, we examined associations between the level of genetically-intact provirus and HIV or HBV clinical infection parameters. We calculated Spearman’s correlations between the levels of these clinical parameters and the infection frequency or genetic diversity of genetically-intact proviruses, as summarized in the Summary Table of Fig. 2.

To investigate whether the levels of HIV production were associated with the frequency of genetically-intact HIV found in cells, we compared the level of plasma HIV RNA to the infection frequency of genetically-intact HIV proviruses. We found that higher levels of plasma HIV RNA were significantly associated with higher levels of genetically-intact HIV provirus in the blood (*P* = 0.02) (Fig. 2A). Increased levels of HIV RNA in the blood were also found to be associated with an increased genetic diversity of genetically-intact provirus in the blood, but this result did not reach statistical significance (*P* = 0.07) (Fig. 2B). However, CD8 and CD4 T cell counts were not found to be associated with the frequency or diversity of genetically-intact HIV provirus (all *P* > 0.05) (Fig. 2C–F). We also measured the total HIV DNA in the liver as previously described (18). Higher levels of HIV DNA in the liver were found to be significantly associated with higher levels of genetically-intact proviruses in the blood (*P* < 0.01) (Fig. 2G). Higher levels of HIV DNA in the liver were also found to be associated with greater genetic diversity of genetically-intact HIV provirus in the blood, although this result did not reach statistical significance (*P* = 0.06) (Fig. 2H).

When examining HBV-related clinical parameters, the levels of HBV DNA in the blood, a marker of HBV replication, were not associated with the infection frequency (Fig. 2I) or the genetic diversity (Fig. 2J) of the genetically-intact HIV proviruses (*P* > 0.05). However, co-infected participants were observed to have one of three patterns: (i) high levels of genetically-intact HIV and high levels of HBV DNA; (ii) low levels of genetically-intact HIV and low levels of HBV DNA; or (iii) low levels of genetically-intact HIV and high levels of HBV DNA (Fig. 2I). No individuals were found to have low levels of HBV DNA with high levels of genetically-intact HIV provirus. Levels of quantitative HBV surface antigen (qHBsAg), another marker of HBV replication, were not significantly associated with the infection frequency (*P* = 0.17) (Fig. 2K) or the genetic diversity of the genetically-intact HIV proviruses (*P* = 0.88) (Fig. 2L). Similar to that observed for the HBV DNA levels, participants were observed to have high levels of both qHBsAg and genetically-intact HIV, low levels of both qHBsAg and genetically-intact HIV, or low levels of genetically-intact HIV with high levels of qHBsAg. There were no participants with a high level of genetically-intact HIV and a low level of qHBsAg. Those HIV-HBV co-infected individuals positive for HBeAg tended to have more genetically-intact HIV provirus than those who were HBeAg negative, but this result did not reach statistical significance (*P* = 0.15) (Fig. 2M). There was no difference between the genetic diversity of the HIV proviruses in the HBeAg-positive or the HBeAg-negative individuals (*P* = 0.65) (Fig. 2N).

### Markers of immune activation and inflammation are associated with increased levels of genetically-intact HIV provirus in untreated HIV-HBV co-infected participants.

**FIG 3:**
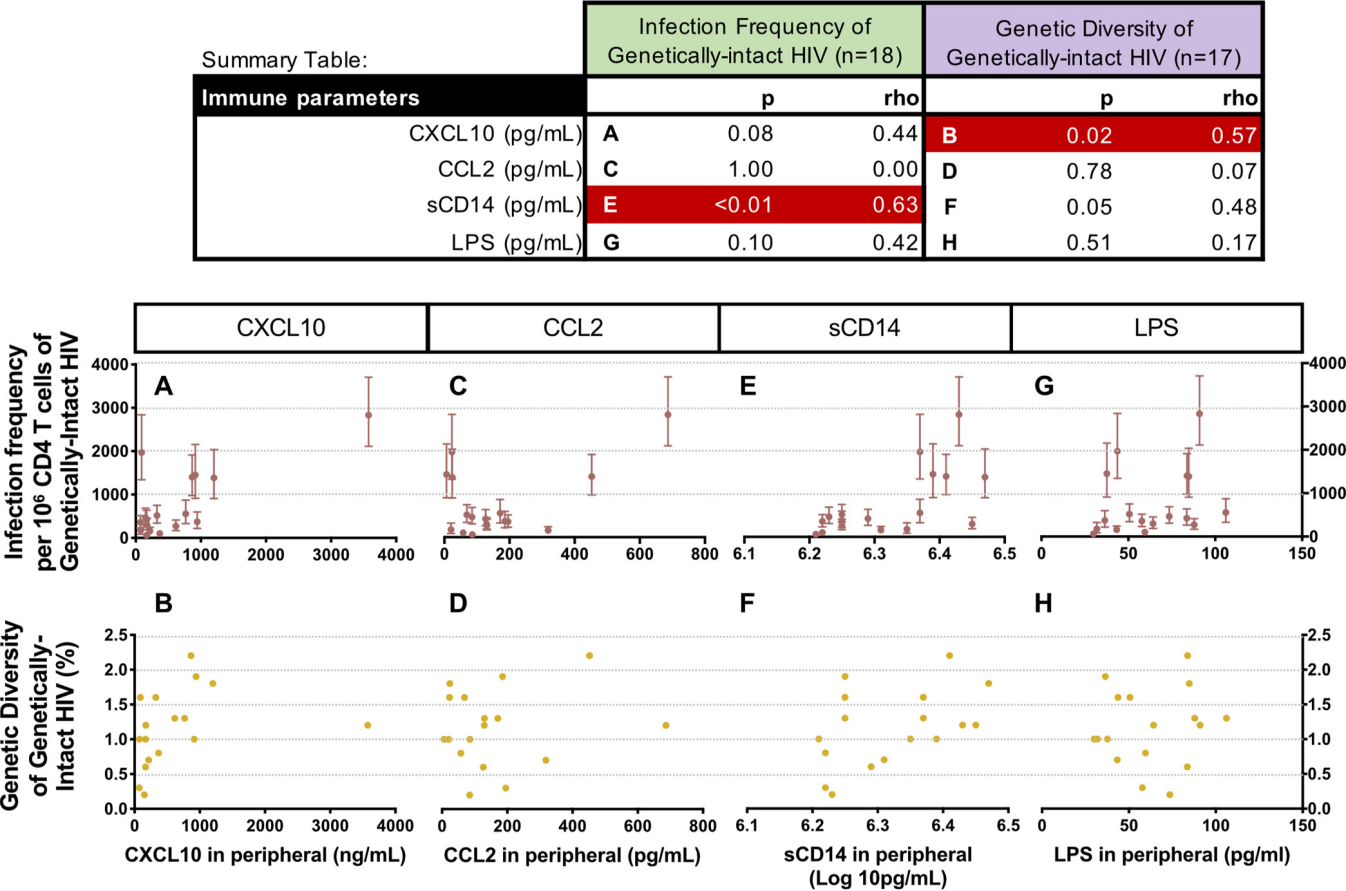
Spearman’s correlation analyses comparing the levels of immune activation and inflammation markers with the infection frequency and the genetic diversity of genetically-intact HIV proviruses (Summary Table: significant associations in red). The association between the infection frequency (maroon) and the diversity (yellow) of genetically-intact HIV proviruses and the levels of immune markers are shown in Fig. 3A–H. The scale of the *x*-axis changes for each immune marker.

Both HIV and HBV infection have previously been shown to be associated with increased immune activation and inflammation (12, 18, 19). Therefore, we investigated whether the levels of genetically-intact HIV proviruses were associated with any markers of immune activation and inflammation. We observed that some markers of immune activation and/or inflammation were associated with both the levels of genetically-intact HIV and the genetic diversity of genetically-intact HIV proviruses, as shown in the Summary Table of Fig. 3.

The levels of the pro-inflammatory cytokine CXCL10 measured in the blood had a positive association with the infection frequency of genetically-intact HIV, and this association approached statistical significance (*P* = 0.08) (Fig. 3A). However, this result was likely to have been largely driven by one participant. Excluding this participant from the analyses reduced the significance further (*P* = 0.22, data not shown). In contrast, higher levels of CXCL10 in the blood were found to be significantly associated with a higher genetic diversity of the genetically-intact HIV proviruses, regardless of whether this participant was included or excluded (*P* = 0.02) (Fig. 3B). Levels of the pro-inflammatory cytokine CCL2 were not significantly associated with either the infection frequency (*P* = 1.00) (Fig. 3C) or the genetic diversity of the genetically-intact HIV proviruses (*P* = 0.78) (Fig. 3D). Soluble CD14 (sCD14), a marker of immune activation, was found to be significantly associated with both the infection frequency (*P* < 0.01) (Fig. 3E) and the genetic diversity of the genetically-intact HIV proviruses (*P* = 0.05) (Fig. 3F). Another marker of immune activation, lipopolysaccharide (LPS), was not significantly associated with either the infection frequency (*P* = 0.10) (Fig. 3G) or the genetic diversity of the genetically-intact HIV proviruses (*P* = 0.51) (Fig. 3H).

**FIG 4:**
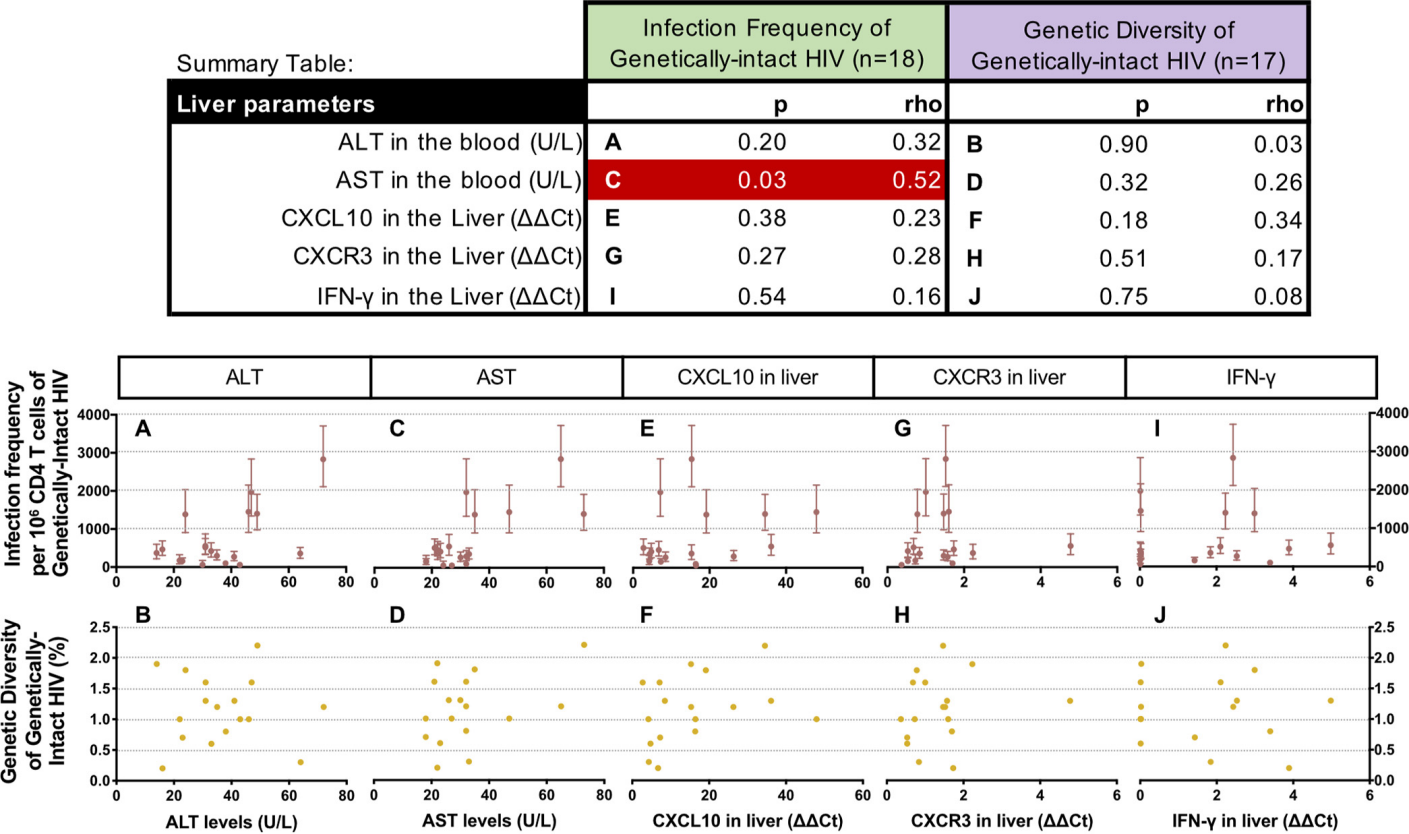
Spearman’s correlation analyses comparing the levels of markers of liver damage and liver inflammation with the infection frequency and the genetic diversity of genetically-intact HIV proviruses (Summary Table: significant associations in red). The association between the infection frequency (maroon) and the diversity (yellow) of genetically-intact HIV proviruses and the levels of markers of liver damage and liver inflammation are shown in Fig. 3A–J. The scale of the *x*-axis changes for each marker of liver damage or liver inflammation.

In addition to measuring markers of inflammation in the plasma, we also measured markers of liver inflammation and liver damage. Spearman’s correlations between the levels of liver inflammation/damage markers and genetically-intact HIV proviruses are shown in the Summary Table of Fig. 4. Previous studies have shown that high levels of the liver enzymes alanine aminotransferase (ALT) and aspartate aminotransferase (AST) in the blood are associated with liver damage, which could be due to HBV infection. Therefore, we measured ALT and AST in the blood as markers of liver damage. There was no significant association between ALT levels and the infection frequency (*P* = 0.20) (Fig. 4A) or the genetic diversity of the genetically-intact HIV proviruses (*P* = 0.90) (Fig. 4B). However, we did observe a significant association between AST levels and the infection frequency of the genetically-intact HIV proviruses (*P* = 0.03) (Fig. 4C) but not the genetic diversity of these proviruses (*P* = 0.32) (Fig. 4D). CXCL10 and CXCR3 mRNA were measured in liver biopsy specimens by qRT-PCR. The expression levels of CXCL10 and its receptor CXCR3 in the liver were not found to be statistically significantly associated with the levels (*P* = 0.38 and *P* = 0.27, respectively) (Fig. 4E and G) or the genetic diversity of genetically-intact HIV in the blood (*P* = 0.18 and *P* = 0.51, respectively) (Fig. 4F and H). In addition, the levels of the inflammatory cytokine IFN-γ measured in liver biopsy specimens by qRT-pCR were not found to be associated with the infection frequency (*P* = 0.54) (Fig. 4I) or the genetic diversity of genetically-intact of genetically-intact HIV proviruses (*P* = 0.75) (Fig. 4J).

### ART-naive HIV-HBV co-infected individuals have more genetically-intact HIV proviruses compared to HIV mono-infected individuals.

**FIG 5:**
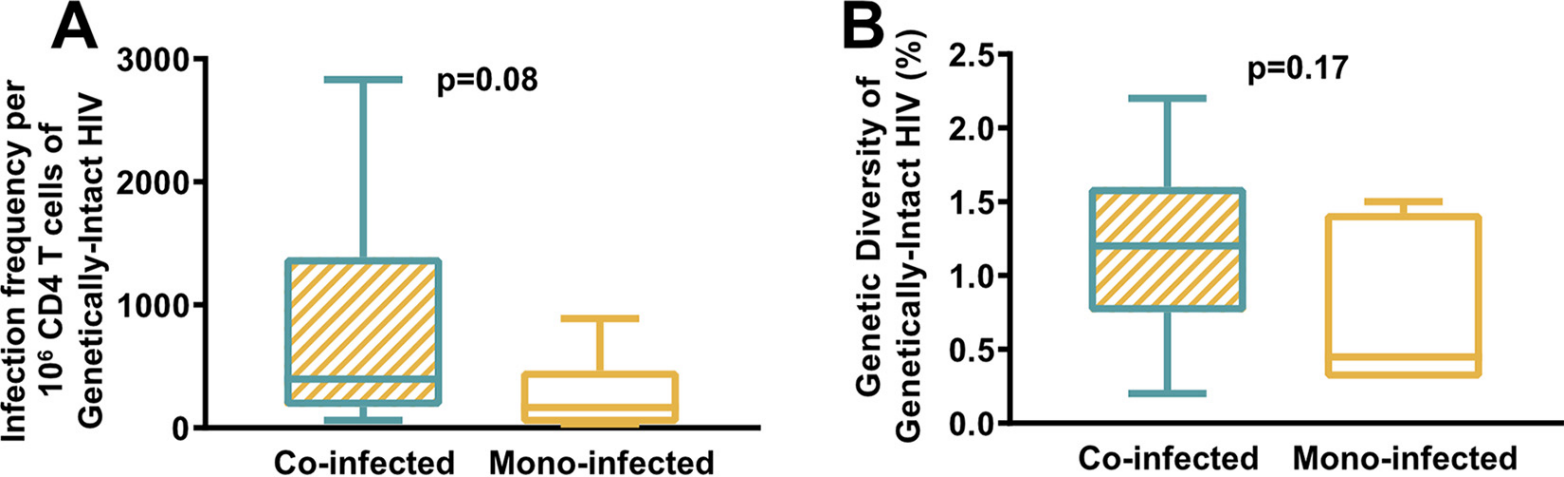
Comparison of HIV CRF01_AE proviruses between HIV-HBV co-infected and HIV mono-infected individuals. (A) Mann-Whitney test comparing the infection frequency of genetically-intact HIV proviruses in co-infected and mono-infected participants. (B) Mann-Whitney test comparing the genetic diversity of genetically-intact HIV proviruses in co-infected and mono-infected participants.

In order to determine how HBV co-infection affects the proviral characteristics of HIV, we compared the sequences of HIV proviruses from the HIV-HBV co-infected cohort with sequences from an HIV mono-infected cohort, prior to treatment initiation. An additional 538 HIV proviruses were sequenced from 6 HIV mono-infected individuals using FLIPS. The infection frequency per 10^6^ CD4 T cells of the genetically-intact HIV proviruses were calculated for all of these participants, as well as the genetic diversity of these genetically-intact HIV proviruses (Fig. 5). HIV-HBV co-infected individuals did have a higher infection frequency of genetically-intact HIV proviruses which were more genetically diverse, but these results did not reach statistical significance (*P* value for infection frequency of genetically-intact proviruses, *P* = 0.08 [Fig. 5A]; *P* value for genetic diversity of genetically-intact proviruses, *P* = 0.17 [Fig. 5B]). It is worth noting that these two participant cohorts were taken from separate clinical studies but were located at the same site. The mono-infected cohort tended to have more female representation (33%) compared to the co-infected cohort (21%), but this difference was not statistically significant (Table S1). There was no difference in age between cohorts (*P* = 0.92) (Fig. S1A). Also, there was no statistically significant difference between the CD4^+^ T cell counts (*P* = 0.15) (Fig. S1B) or the plasma HIV RNA levels (*P* = 0.06) (Fig. S1C) between these two participant cohorts.

Although there were no significant differences in sex and age between the mono-infected and co-infected cohorts, we examined whether any of these factors would be confounders in our correlation analyses. Age was determined to have no correlation with the infection frequency or the genetic diversity of genetically-intact HIV across either the co-infected cohort or the mono-infected cohort (Fig. S1D and E). Male and female participants were not found to have any significant differences between the levels of genetically-intact proviruses or between the genetic diversity of these genetically-intact proviruses (Fig. S1F and G).

### Comparisons between the pre-therapy and on-therapy HIV proviral landscape.

**FIG 6:**
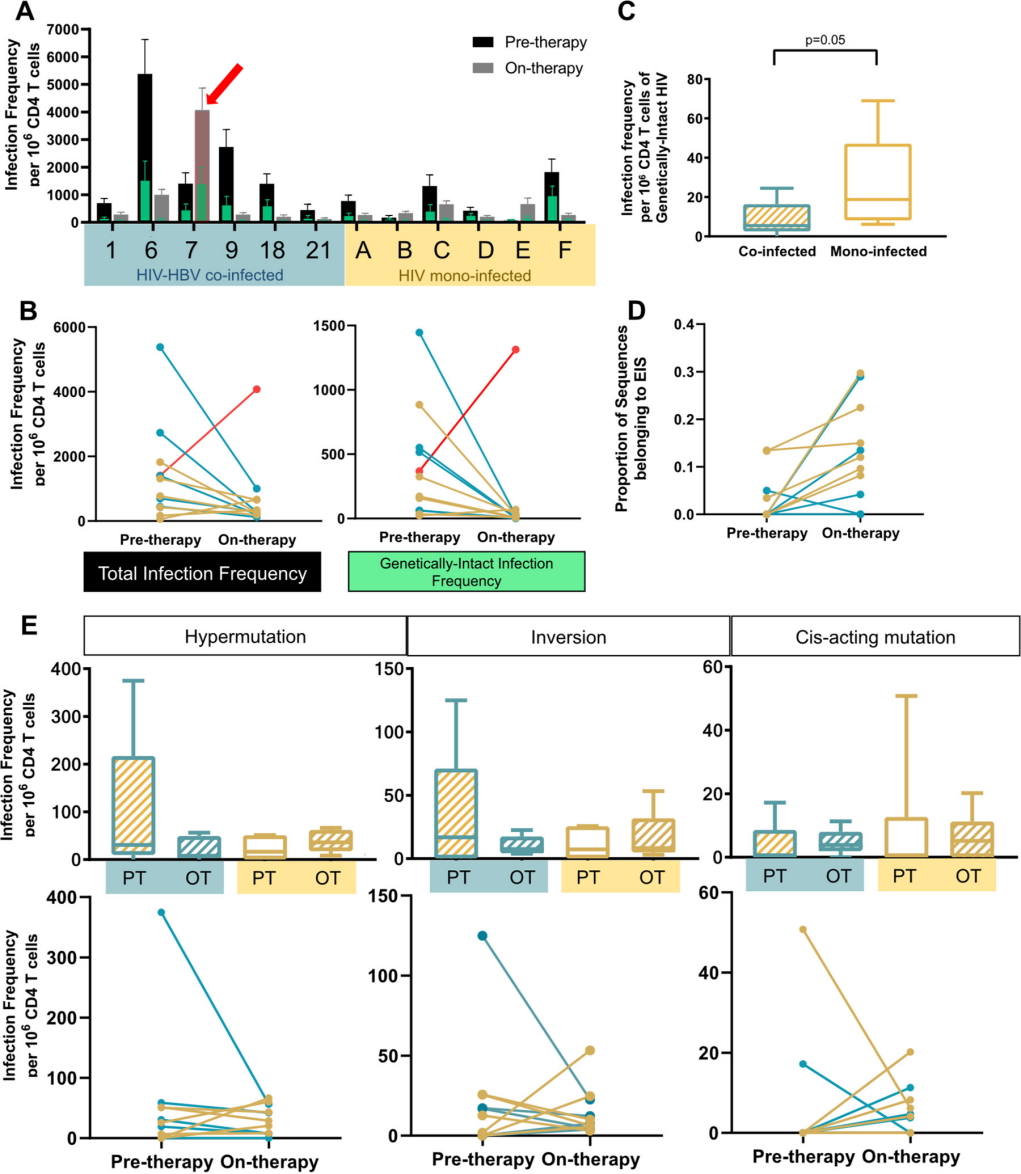
Comparison of HIV proviruses in HIV-HBV co-infected and HIV mono-infected participants after 3 years of antiretroviral therapy. (A) Infection frequency of HIV per 10^6^ CD4 T cells from both pre-therapy and on-therapy samples from 6 mono-infected participants, where the frequency of genetically-intact HIV (in green) may also be viewed as a proportion of the total HIV frequency. The red arrow indicates a participant that was still viremic at the on-therapy sampling time point. (B) Change in the infection frequency of HIV proviruses pre-therapy and on-therapy, sequenced from HIV-HBV co-infected participants (blue) and HIV mono-infected participants (yellow). In red is a co-infected individual who was still viremic at the time of on-therapy sampling. Panels C to E exclude this viremic participant. (C) Comparison of the infection frequency of genetically-intact HIV proviruses between co-infected individuals and mono-infected individuals after 3 years of ART. Statistical significance was calculated with a Mann-Whitney test. (D) Change in the proportion of sequences belonging to an expansion of identical sequences (EIS) in co-infected participants (blue) and mono-infected participants (yellow). (E) Change in the infection frequency of HIV proviruses with different defects in pre-therapy (PT) and on-therapy (OT) sequenced from HIV-HBV co-infected participants (blue) and HIV mono-infected participants (yellow). Mixed effects modelling was used to calculate the *P*-value. The top row shows the comparison of infection frequencies between groups, while the bottom row shows the change over time as paired data.

We then examined whether there were any differences between the HIV proviruses in the HIV-HBV co-infected and HIV mono-infected individuals after at least 2 years of ART. As expected, the infection frequency of genetically-intact HIV proviruses decreased in all participants, except for one participant (highlighted with a red arrow in Fig. 6A) who was not adherent to ART at the time of the on-therapy sample collection. For all subsequent analyses, the data obtained from the participant who was still viremic during therapy were excluded. While the infection frequency of total HIV proviruses decreased after therapy in all HIV-HBV co-infected individuals, 2 of the 6 HIV mono-infected individuals had an increased overall HIV infection frequency after at least 2 years of ART (Fig. 6A and B). This was surprising, but this could be attributed to differences in sampling, as fewer sequences were obtained from the pre-therapy samples (*n* = 23 and *n* = 31) compared to the on-therapy samples (*n* = 52 and *n* = 40, respectively) from these two participants. Furthermore, in those mono-infected individuals who have lower HIV infection frequencies before therapy, any decrease in HIV infection frequency after therapy initiation may be less obvious compared to the decreases observed in co-infected individuals (Fig. 5A). However, ART would naturally decrease the infection frequency of HIV proviruses (15).

We then compared the the infection frequency of genetically-intact HIV proviruses between the HIV-HBV co-infected cohort and the HIV mono-infected cohort after at least 2 years of ART. In contrast to what was observed in the pre-therapy samples, the mono-infected individuals had higher levels of genetically-intact HIV proviruses than did the co-infected individuals (*P* = 0.05) (Fig. 6B and C). The proportion of sequences belonging to an expansion of identical sequences (EIS), defined as ≥2 genetically identical sequences, was significantly increased for both cohorts after at least 2 years of therapy (*P* = 0.01) (Fig. 6D).

Next, we examined changes in the infection frequencies of three types of genetic defects in the HIV genome between the pre-therapy and on-therapy time points. Interestingly, the HIV mono-infected individuals had significantly increased levels of hypermutated proviruses during ART (*P* = 0.04) (Fig. 6E). However, in the HIV-HBV co-infected cohort, we observed a significant decrease in hypermutation after 3 years of ART (*P* = 0.02) (Fig. 6E). Overall, there was a statistically significant difference in how ART affects the level of hypermutated HIV proviruses between the two cohorts (*P* = 0.02). There was no statistically significant difference between the co-infected and mono-infected cohorts regarding the levels of other genetic defects within the HIV proviruses, such as inversions and cis-acting mutations.

## DISCUSSION

In order to further understand the impact of HBV co-infection on HIV replication and reservoir formation, we examined associations between HBV infection parameters and the levels of genetically-intact HIV proviruses in HIV-HBV co-infected individuals. We observed that specific HIV clinical parameters were linked with the level of genetically-intact HIV in ART-naive HIV-HBV co-infected individuals. In comparing HIV-HBV co-infected and HIV mono-infected individuals before therapy, the HIV mono-infected individuals were found to have a lower frequency of genetically-intact HIV than did the HIV-HBV co-infected individuals, although this result did not reach statistical significance. These mono-infected individuals had significantly higher CD4^+^ T cell counts and significantly lower plasma HIV RNA levels than did the HIV-HBV co-infected individuals. However, interestingly, after 3 years on ART, the levels of genetically-intact HIV in the mono-infected individuals were higher than those observed in the co-infected individuals. In this study, CD4^+^ T cell counts were not found to be significantly associated with levels or genetic diversity of genetically-intact HIV provirus in HIV-HBV co-infected participants, pre-therapy. Previous studies have also observed lower CD4^+^ T cell counts and higher HIV RNA levels in HIV-HBV co-infected individuals (8, 20, 21). In addition, studies have found that lower CD4^+^ T cell counts are strongly associated with higher levels of plasma HIV RNA (22). Therefore, this association between HBV clinical parameters and levels of genetically-intact HIV provirus, pre-therapy, in HIV-HBV co-infected individuals indicates that HBV co-infection may be responsible for the lower CD4^+^ T cell count in the co-infected individuals, which would mean that this is not a confounding factor in the analyses. In order to confirm this, we measured established markers of HBV replication, such as HBV DNA levels, HBeAg status, and HBsAg levels (2, 23). However, we were unable to find a significant association between HBV replication and the level of genetically-intact HIV in the co-infected individuals when using any of these markers of HBV replication.

As HIV pathogenesis is associated with increased immune activation, we examined correlations between certain markers of immune activation/inflammation and the levels and diversity of genetically-intact HIV in the co-infected individuals. We identified one marker of immune activation, sCD14, which significantly correlated with the level of genetically-intact HIV proviruses. sCD14 is a marker of monocyte activation, and it acts as a marker of LPS-induced microbial translocation (24, 25). Increased levels of sCD14 are a predictor of mortality in HIV infection (26), as it is also associated with increased systemic immune activation (27). Furthermore, a study in Guangzhou found that HIV-HBV co-infected individuals have higher levels of sCD14 than do HIV mono-infected individuals (14). In agreement with this study, we observed a significant correlation between sCD14 levels and levels of genetically-intact HIV within HIV-HBV co-infected individuals, suggesting that the higher levels of immune activation in these individuals contributed to their increased level of genetically-intact HIV proviruses compared to those of the mono-infected participants. Although causation cannot be determined in this study, ongoing viral replication during untreated infection could also contribute to enhanced immune activation.

We also observed another marker of inflammation, CXCL10, to be significantly associated with the diversity of genetically-intact HIV, and this marker had a positive association with the infection frequency of genetically-intact HIV proviruses in the co-infected individuals. Recent studies in the context of HIV mono-infection have shown that higher levels of CXCL10 in the blood were associated with more rapid HIV disease progression and lower CD4^+^ T cell counts, in addition to being a reliable marker for predicting the progression to AIDS (19, 28, 29). The increased genetic diversity of HIV has also been linked to poorer outcomes in HIV-positive individuals (30). Interestingly, higher levels of CXCL10 have also been linked to increased HBV DNA, HBsAg, ALT, and AST levels (31). Further studies would be required to elucidate whether it is the HIV or HBV infection that increases CXCL10 levels in co-infected individuals compared to mono-infected individuals. This could be a critical link in determining how HIV-HBV co-infection leads to such a poor prognosis compared to that of mono-infection. Of note, a study of HIV-HBV co-infected individuals compared to HBV mono-infected individuals found that levels of sCD14 and CXCL10 were also significantly higher in HIV-HBV co-infected individuals compared to HBV mono-infected individuals (12). Overall, this suggests that immune activation leading to microbial translocation is a potential mechanism by which HIV-HBV co-infection results in poorer outcomes compared to those of individuals infected with either virus alone.

Our study found that there was an association between the levels of the liver enzyme AST and the infection frequency of genetically-intact HIV, which indicates an association between liver damage and HIV replication. This finding is supported by previous studies, which have found associations between increased levels of ALT in HIV-HBV co-infection compared to HIV mono-infection (8). Chronic liver damage and its long-term complications can only be partially stopped by ART, and these can also contribute to the poorer outcome of co-infected individuals (32). Interestingly, we were only able to find a significant correlation between AST levels and genetically-intact HIV, as ALT levels and genetically-intact HIV displayed a weaker correlation. A study that focused on associations between HIV RNA loads and levels of liver aminotransferases found similar results but suggested that this pattern could be due to the injury of other tissues, such as muscle, lung and kidney, along with liver damage, due to the cytopathic nature of an HIV infection (33). Although we found a significant association between levels of HIV DNA in the liver and levels of genetically-intact HIV provirus in the peripheral blood, cells obtained from the liver biopsy specimen were not sorted before DNA extraction, so the sample could potentially be contaminated with PBMCs or infiltrating CD4^+^ T cells. The HIV DNA from these contaminating PBMCs could be driving the association between levels of HIV DNA in the liver and levels of genetically-intact HIV in the blood. However, unlike in the peripheral blood, the levels of CXCL10 expression in the liver were not significantly correlated with levels or diversity of genetically-intact provirus in the blood, indicating that liver inflammation may not be associated with increased HIV replication.

To observe the effects of HIV-HBV co-infection on HIV reservoir formation, we conducted the first study to characterize HIV proviruses after 3 years on ART in HIV-HBV co-infected individuals compared to HIV mono-infected individuals. Interestingly, while we observed significantly higher levels of genetically-intact HIV proviruses in HIV-HBV co-infected individuals, pre-therapy, there was a trend of higher levels of genetically-intact HIV proviruses in the mono-infected individuals after 3 years of ART. This could be due to the lower number of participants sampled on-therapy; increasing the number of on-therapy participants would provide a more accurate estimation of this trend. When examining specific genetic defects in the HIV provirus, it is interesting that there was a trend of higher infection frequency of defective provirus in the mono-infected cohort after 3 years on ART, particularly with the hypermutation defect. Hypermutation of the HIV genome is known to be facilitated by the host APOBEC3G enzyme, although viral proteins such as Vif aim to block this interaction (34). APOBEC3G has also been found to play a role in combating HBV infection, yet it is unknown whether HBV has evolved any mechanism to inhibit this antiviral enzyme (35). Our finding of more hypermutated HIV proviruses in mono-infected participants during therapy suggests that HIV-HBV co-infection could potentially inhibit the effects of APOBEC3G more so than HIV infection alone.

We observed an increase in identical HIV proviral sequences after 3 years on ART, suggesting the importance of cellular proliferation in maintaining the HIV reservoir, which has been shown previously (17, 36, 37). However, there does not appear to be any difference in the rate of cellular proliferation in the co-infected cohort compared to the mono-infected cohort, suggesting that HBV infection does not lead to increased levels of cellular proliferation. Furthermore, whether HBV co-infection has any impact on HIV replication or reservoir formation is controversial. Most of the studies investigating this topic tend to be clinical population studies that assess mortality rates and causes, which ultimately provide varying conclusions as to whether HBV co-infection speeds the progression to AIDS (10, 38–41).

There are a few limitations to this study. We use the term genetically-intact to describe HIV proviruses that are lacking any known defects in their genome. However, this is not the same as HIV proviruses that have been deemed to be replication competent. While FLIPS is able to provide vast amounts of genomic data, our theoretical knowledge is currently not able to definitively conclude whether a virus would be able to replicate just from knowing its sequence. However, we believe it to be the best compromise for inferring the replicative potential of a virus, as it is simply not feasible to grow all 567 genetically-intact viruses that were sequenced as a part of this study. FLIPS is also unable to differentiate between integrated proviruses and 2LTR circles. However, we have previously found few 2LTR circles present in samples, and the participants in this study were not treated with raltegravir, so we believe that they should not contaminate our results (16). On the other hand, FLIPS is able to provide more information about the viability of the virus compared to standard quantification measures, such as HIV DNA levels or HIV RNA levels. It is also important to note that the co-infected and mono-infected cohorts were recruited as part of different clinical studies; however, there was no statistically significant difference between these cohorts in age distribution, sex distribution, or plasma HIV RNA and CD4^+^ T cell counts. In the literature, chronic HIV infection is associated with greater proviral genetic diversity, so the stage of HIV infection during sample collection could have an impact on the genetic diversity of the proviruses sequenced (42–45). Unfortunately, the time of HIV infection for both cohorts is unknown, meaning that the stage of infection during sample collection was uncertain. As we were only able to obtain samples from 6 HIV-HBV co-infected participants after 2 years of therapy, we were unable to determine whether the correlations between markers of immune activation or inflammation were also associated with levels or diversity of genetically-intact HIV proviruses in these treated participants. Furthermore, only 6 HIV mono-infected participants were enrolled in this study, so we were unable to observe a statistically significant difference in the levels of genetically-intact HIV provirus between the HIV-HBV co-infected and HIV mono-infected cohorts before therapy.

Our study took a novel approach to investigate the impact of HBV co-infection on HIV infection by focusing our studies at a molecular level as well as by investigating indirect associations between the two viruses. We propose a potential mechanism by which co-infection leads to a poorer prognosis. Our data suggest that HBV co-infection may drive a more activated and pro-inflammatory immune profile, which is in turn associated with higher HIV viremia and an increased number of genetically-intact HIV proviruses in peripheral blood cells. However, further studies will be necessary to examine this further, as correlations do not provide any indication as to which parameter is driving the result. A better understanding of the mechanisms behind the increased mortality rates of co-infected individuals will assist in developing better therapies to mitigate this important issue.

## MATERIALS AND METHODS

### Participants.

Whole blood draws were collected from 18 HIV-HBV co-infected and 6 HIV mono-infected participants from Bangkok, Thailand prior to ART initiation (Table S1). These participants were a subset of the participants enrolled in a related study (18). These participants were all infected with HIV CRF01_AE. All participants co-infected with HCV were excluded from this study. The duration of infection prior to ART initiation is unknown for all participants. Of the 18 HIV-HBV co-infected participants, 6 returned for another whole blood draw after an average of 3.1 years on ART (range: 2.0 to 3.8 years). Meanwhile, all 6 of the HIV mono-infected participants returned for another whole blood draw after an average of 6.4 years on ART (range: 5.5 to 8.6 years).

### Sample processing and quantification of viral infection parameters, markers of immune activation and inflammation and liver inflammation.

The quantification of HIV DNA from purified peripheral blood CD4^+^ T cells, normalized to cell number by the quantification of the housekeeping gene CCR5, was conducted as previously described (18). HBV DNA levels and qHBsAg levels were quantified as previously described (18). The cytokines that were used as markers of immune activation and inflammation (CXCL10, CCL2, sCD14, and LPS) were quantified using ELISA or multiplex as previously described (18).

Liver samples were obtained from liver punch biopsy specimens and processed through lysis and homogenization with the Qiagen Qiashredder column as previously described (18). DNA quantification of HIV was conducted as described for the CD4 T cells. Quantification of the mRNA of CXCL10 and CXCL3 were performed using qRT-PCR as previously described, with relative amounts of PCR product determined using the delta-delta Ct method (18).

### Full-Length Individual Proviral Sequencing of HIV.

The Full-Length Individual Proviral Sequencing (FLIPS) assay was used to sequence near full-length HIV proviruses from the extracted DNA of the PBMCs from these HIV-HBV co-infected and HIV mono-infected participants (16). In brief, extracted DNA was diluted so that approximately 30% of the wells were positive, indicating that a nested PCR would most likely be performed on a single copy of HIV, which would prevent a bias of amplification toward shorter HIV sequences. HIV CRF01_AE-specific primers were used to amplify the proviruses: PCR Round 1 Forward (BlouterF) 5′-AAATCTCTAGCAGTGGCGCCCGAACAG-3′, PCR Round 1 Reverse (BlouterR) 5′-TGAGGGATCTCTAGTTACCAGAGTC-3′, PCR Round 2 Forward (AE275F) 5′-ACAGGGACYYKAAAGYGAAAG-3′, PCR Round 2 Reverse (AE280R RYY) 5′-CTAGTTACCAGAGTCCTRACACARAYG-3′. Cycling conditions and reagent concentrations for PCR Round 1 and PCR Round 2 were as previously described (16). Amplicons were cleaned and quantified for library preparation and next-generation sequencing on the Ilumina MiSeq platform (Australian Genome Research Facility, Sydney, Australia).

### Data analysis.

HIV proviral contigs were assembled de novo using a CLC Genomics Workbench pipeline (16). In brief, the forward and reverse sequences of each read for each sample were paired, and the index adaptor sequences were trimmed. Ambiguous nucleotides, two 5′ terminal nucleotides and 15 nucleotides at the 3′ terminal end, were also removed as a part of the workflow. The trimming of contigs was also conducted based on a cutoff of 30 for the Phred score, indicating 99.9% accuracy. Overlapping regions of forward and reverse reads were merged to extend read lengths, followed by the subsampling of 10,000 paired reads for the de novo assembly of contigs. The remaining reads were then mapped to these contigs. The amplification of more than one HIV proviral template, which can be determined by uneven coverage across the HIV genome sequenced or by an inability to combine multiple contigs of HIV, was termed a “mixture” and was excluded from the analyses. HIV proviruses from each participant were aligned using the E-INS-I model on MAFFT version 7 (46). Alignments were manually checked in Molecular Evolutionary Genetics Analysis (MEGA). The Gene Cutter and Hypermut tools with default settings (http://www.hiv.lanl.gov) were used to determine whether the proviruses were defective due to stop codons, frameshifts, or G→A hypermutation. Proviruses were also scanned by eye for any mutations or deletions in Stem Loop 2, specifically the Major Splice Donor (MSD) in the cis-acting region of the HIV genome (position 740 to 750 on HXB2 [47]). Elimdupes (Los Alamos HIV Database) was used to identify expansions of identical sequences (100% identity).

### Phylogenetic analyses.

Phylogenetic analyses were performed with MEGA 7. In order to determine whether there was contamination between the participant samples or with the laboratory strain (BaL), neighbor-joining trees were generated from all of the aligned full-length sequences for each participant.

When investigating the genetic diversity of the HIV proviruses in each participant’s samples, pairwise distance was calculated with MEGA 7. The p-distance model was used with a bootstrap value of 500 and was Gamma distributed with Invariant sites settings. The p-distance model calculates the distances between two sequences on a phylogenetic tree as a fraction of the different positions between them. In order to determine the overall diversity between all of the HIV sequences within a participant sample, the average pairwise distance was calculated and expressed as a percentage difference.

### Statistics.

Confidence intervals for HIV infection frequencies were determined by using the binomial test (“binom.test” function in R) and scaling this proportion to the million cells units. Spearman’s rank correlations were calculated in R, a language and environment for statistical computing, using approximate tests. Pre-therapy and on-therapy infection frequencies of genetic defects with adjustment for HIV-HBV co-infection or HIV mono-infected groups were compared with a mixed logistic regression with a random effect for the intercept and the pre/on-therapy effect in R using the “lme4” library. Mann-Whitney tests for unpaired data and Wilcoxon matched pairs signed-rank tests for paired data were performed in GraphPad Prism 8.4.

### Study approval.

This study was approved by the institutional review board at the Western Sydney Health Department for the Westmead Institute for Medical Research, The Alfred Hospital Ethics Committee (76/12), and the institutional review board of the Faculty of Medicine, Chulalongkorn University (341/55). Written informed consent was obtained from all participants.

### Data availability.

All sequences analyzed in this study have been deposited in GenBank under accession numbers ON901934-ON903151 and ON862980-ON863357.
