# Supplementary material for: Markers of Immune Activation and Inflammation Are Associated with Higher Levels of Genetically-Intact HIV in HIV-HBV Co-Infected Individuals
Source: J Virol. 2022 Aug 2;96(16):e00588-22. doi: 10.1128/jvi.00588-22 (PMC9400477; doi:10.1128/jvi.00588-22)
Supplement: Supplemental file 1 — Table S1 and Fig. S1. Download jvi.00588-22-s0001.pdf, PDF file, 0.7 MB [file jvi.00588-22-s0001.pdf]

Supplementary Materials

Supplementary Table 1: Individual participant characteristics

ID	Sex	HBV co-infection status	ART status	Cells used	HIV sequences obtained	Intact HIV sequences obtained	Inversions	Large Internal Deletions	Early Stop Codon	Frame-shift	Hyper-mutation	Cis-acting Mutation	Average p-distance of genetically-intact
1	Male	Co-infection	pre-therapy	118833	68	7	2	52	0	0	7	0	0.01
2	Male	Co-infection	pre-therapy	11260	19	4	1	13	0	0	1	0	N/A
3	Male	Co-infection	pre-therapy	15087	63	27	0	30	0	2	3	1	0.016
4	Male	Co-infection	pre-therapy	56835	28	9	0	19	0	0	0	0	0.01
5	Male	Co-infection	pre-therapy	73476	53	19	0	29	1	2	1	1	0.012
6	Male	Co-infection	pre-therapy	16012	73	22	2	40	1	2	6	0	0.01
7	Female	Co-infection	pre-therapy	45533	57	16	2	37	1	0	1	0	0.019
8	Male	Co-infection	pre-therapy	52162	57	21	1	22	1	2	10	0	0.006
9	Male	Co-infection	pre-therapy	32578	67	16	0	49	0	1	1	0	0.013
10	Male	Co-infection	pre-therapy	213863	38	16	3	15	0	1	2	1	0.052
11	Male	Co-infection	pre-therapy	18718	70	24	0	42	2	2	0	0	0.018
12	Female	Co-infection	pre-therapy	55343	62	26	0	28	0	1	6	1	0.002
13	Male	Co-infection	pre-therapy	22614	58	38	1	13	3	2	1	0	0.013
14	Male	Co-infection	pre-therapy	157511	34	15	1	16	1	1	0	0	0.008
15	Male	Co-infection	pre-therapy	26674	70	33	0	34	0	1	2	0	0.022
16	Female	Co-infection	pre-therapy	34347	58	45	0	9	0	1	3	0	0.012
17	Male	Co-infection	pre-therapy	137126	44	20	1	20	1	1	1	0	0.007
18	Male	Co-infection	pre-therapy	52258	60	25	0	33	0	1	1	0	0.016
19	Female	Co-infection	pre-therapy	77808	65	20	2	37	0	1	4	1	0.013
20	Male	Co-infection	pre-therapy	76273	66	25	0	37	1	1	2	0	0.003
21	Male	Co-infection	pre-therapy	58124	21	3	1	16	0	0	0	1	N/A
1	Male	Co-infection	on-therapy	213644	48	0	1	37	0	0	9	1	N/A
6	Male	Co-infection	on-therapy	121311	48	2	2	38	0	0	5	1	N/A
7	Female	Co-infection	on-therapy	29679	54	23	1	26	1	1	2	0	N/A
9	Male	Co-infection	on-therapy	263617	52	2	2	44	0	1	2	1	N/A
18	Male	Co-infection	on-therapy	258979	28	1	1	22	0	0	3	1	N/A
21	Male	Co-infection	on-therapy	405991	38	2	5	31	0	0	0	0	N/A
A	Female	HIV mono-infection	pre-therapy	80490	50	12	0	29	0	0	4	5	1.5
A	Female	HIV mono-infection	on-therapy	324818	52	2	1	33	0	0	14	2	N/A
B	Male	HIV mono-infection	pre-therapy	38831	38	20	0	17	0	0	1	0	0.3
B	Male	HIV mono-infection	on-therapy	198157	49	3	2	38	0	0	3	3	N/A

C	Female	HIV mono-infection	pre-therapy	153377	43	12	1	29	0	0	1	0	0.3
C	Female	HIV mono-infection	on-therapy	379985	49	6	2	24	0	0	13	4	N/A
D	Male	HIV mono-infection	pre-therapy	141479	31	12	1	16	1	1	0	0	0.3
D	Male	HIV mono-infection	on-therapy	362589	40	5	4	25	0	1	5	0	N/A
E	Male	HIV mono-infection	pre-therapy	551027	47	27	1	18	0	0	1	0	0.6
E	Male	HIV mono-infection	on-therapy	75093	64	8	2	44	0	0	9	1	N/A
F	Male	HIV mono-infection	pre-therapy	153376	23	5	0	17	1	0	0	0	1.4
F	Male	HIV mono-infection	on-therapy	379984	52	2	5	39	0	0	5	1	N/A

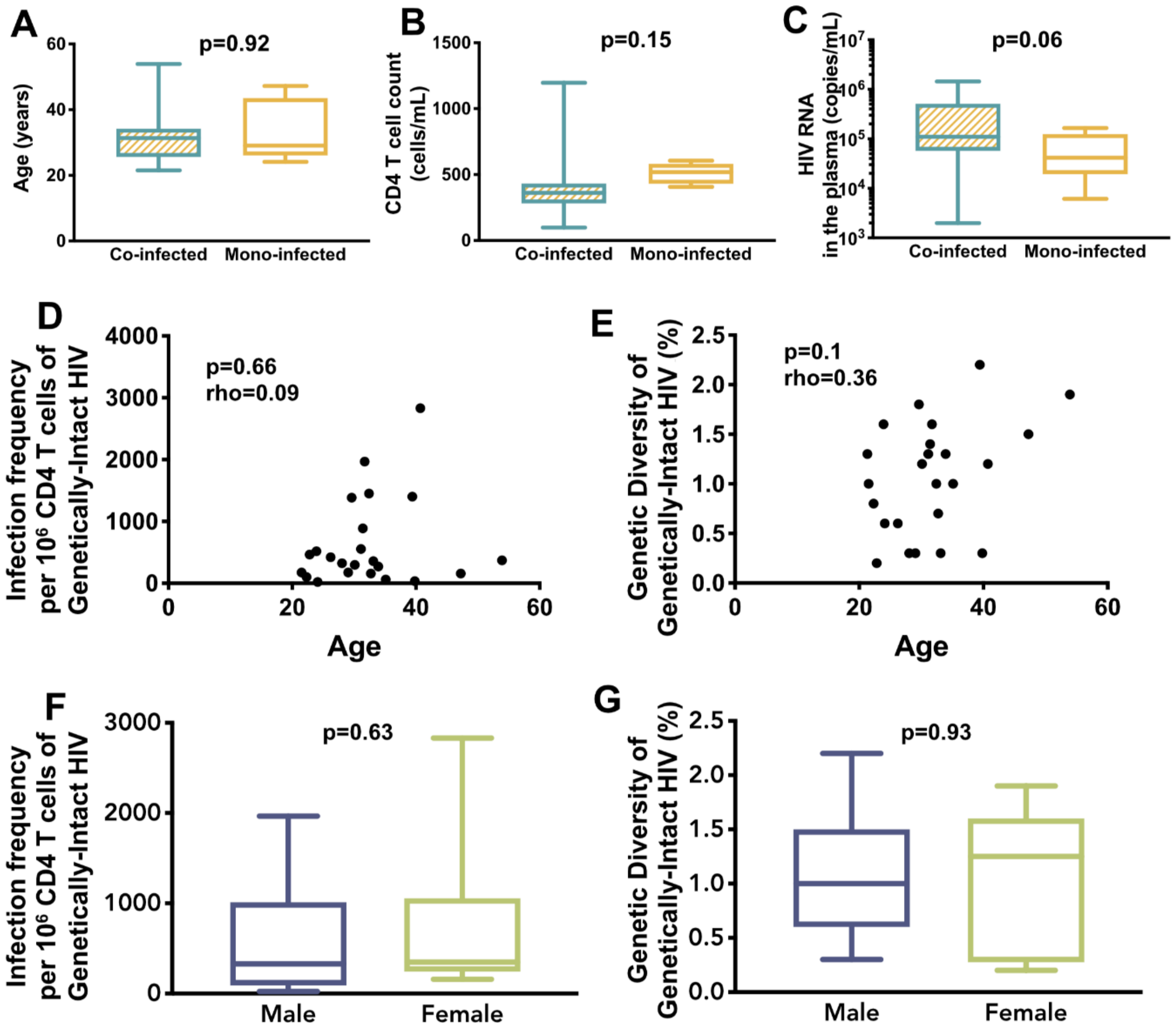

Supplementary Figure 1: Comparison of the age (A), CD4+ T cell counts (B) and HIV RNA levels (C) between the HIV-HBV co-infected and HIV mono-infected cohort. Mann-Whitney tests were performed to determine whether these cohorts differed significantly across these three parameters. Spearman correlation tests on the relationship between the age of the individual and D) the infection frequency of genetically-intact HIV proviruses and E) the genetic diversity of genetically-intact HIV proviruses sequenced from that individual. F) Mann-Whitney test comparing the infection frequency of genetically-intact HIV proviruses between male and female participants, regardless of HBV infection status. G) Mann-Whitney test comparing the genetic diversity of genetically-intact HIV proviruses between male and female participants, regardless of HBV infection status.
